# Nuclear protein of the testis midline carcinoma of the thorax

**DOI:** 10.1093/jjco/hyac033

**Published:** 2022-03-23

**Authors:** Ayae Saiki, Keita Sakamoto, Yuan Bee, Takehiro Izumo

**Affiliations:** Department of Respiratory Medicine, Japanese Red Cross Medical Center, Tokyo, Japan; Department of Respiratory Medicine, Japanese Red Cross Medical Center, Tokyo, Japan; Department of Pathology, Japanese Red Cross Medical Center, Tokyo, Japan; Department of Respiratory Medicine, Japanese Red Cross Medical Center, Tokyo, Japan

## Abstract

Nuclear protein of the testis (NUT) midline carcinoma (NMC) is a rare tumor that typically presents in the head, neck, and chest region. NMC is characterized by rearrangement of the *NUTM1* gene. It mainly affects children and young adults and is rapidly progressive and lethal. Reportedly, the prognoses of NMCs of the head and neck improve following aggressive initial surgical resection +/− postoperative chemoradiotherapy (CRT) or radiotherapy (RT). However, as NMC of the thorax was identified later, treatments to improve its prognosis are yet to be identified. Our review reveals that NMC is an extremely rare cancer, and most patients remain undiagnosed. Furthermore, this review outlines the clinical characteristics of NMC of the thorax and the prospects for its treatment.

## Introduction

### Discovery of t (15,19) cancer

Nuclear protein of the testis (NUT) midline carcinoma (NMC) is a rare tumour that typically presents in the head, neck and chest region. It primarily affects children and young adults and is rapidly progressive and lethal. NMC is characterized by rearrangement of the *NUTM1* gene, which generally results from a translocation of *NUTM1* on chromosome 15q14 and *BRD4* on chromosome 19p13 (1).

Thymic carcinoma harbouring a t(15;19)(q14;p13) translocation was first reported in Japan in 1991 (2). Thereafter, three more cases with a similar translocation were reported in the 1990s and early 2000s (3–5). In 2018, French acquired the Ty-82 cell line that was established from the original Japanese case and created the cell line 00–143. Using these cell lines, he identified the fusion oncogene BRD4–NUT resulting from the t(15;19) translocation (6). Following the screening of poorly differentiated neoplasms in children and young adults, 11 more cases with NUT gene rearrangement were identified. In 2004, these cases were characterized clinically, pathologically and genetically as a disease entity, namely, ‘cancer with t(15;19) translocation’ (7). NMC was first included in the World Health Organization Classification of Tumor in 2015 (8).

### NMC registry

The International NMC registry was formed by Brigham and Women’s Hospital, Dana-Farber Cancer Institute, Boston Children’s Hospital and Harvard Medical School to increase NMC awareness. In 2019, Chau et al. (9) reported the clinical characteristics of 141 cases included in this registry.

The NMC registry included 67 males and 74 females, and the median age at NMC onset was 23.6 years. The primary site was the thorax in 50% of patients, head and neck in 41% and bone and soft tissue in 6% of patients. At the time of NMC diagnosis, metastasis was identified in 50% of the patients. Most of the cases were BRD4-NUT fusion (70%), followed by BRD3-NUT (13%) and NSD3-NUT (5%) (Table 1). The median survival time was 6.5 months.

**Table 1:** Clinical features of NUT midline carcinoma registry

Registry		Number of patient		Percentage (%)	
**Total number of patient**		141			
**Survival time (month)**		6.5			
**Age (years old)**		23.6(18 days–80 years)			
<18		47		33	
≧18		77		55	
Unknown		17		12	
**Sex**					
Male		67		48	
Female		74		52	
**Primary tumour site**					
Thoracic		71		50	
Head and neck		58		41	
Bone and soft tissue		9		6	
Other		2		1	
Unknown		1		0.7	
**Metastasis**					
Yes		71		50	
No		42		30	
Unknown		28		20	
**Chemotherapy**					
Yes		105		74	
No		12		9	
Unknown		24		17	
**Radiation**					
Yes		85		60	
No		32		23	
Unknown		24		17	
**Surgery**					
Yes		60		43	
No		58		41	
Unknown		24		18	
**Gene fusion**					
BRD4-NUTM1		99		70	
BRD3-NUTM1		19		13	
NSD3-NUTM1		7		5	
ZNF532-NUTM1		1		0.7	
ZNF592-NUTM1		1		0.7	
Unknown		9		6	

**Table 2:** Clinical features of NUT of the thorax investigation

NMC of the thorax		Number of patient		Percentage(%)	
**Total number of patient**		65			
**Survival time (month)**		6.75			
**Age (years old)**		30.0 (5–70 years)			
<18		12		18	
≧18		53		82	
Unknown		0		0	
**Sex**					
Male		34		52	
Female		30		46	
Unknown		1		2	
**Primary tumour site**					
Lung		40		62	
Mediastinum		19		29	
Thymus		2		3	
Unknown		4		6	
**Metastasis**					
Yes		29		44	
No		20		31	
Unknown		16		25	
**Chemotherapy**					
Yes		43		66	
No		6		9	
Unknown		16		25	
**Radiation**					
Yes		26		40	
No		20		31	
Unknown		19		29	
**Surgery**					
Yes		14		22	
No		32		49	
Unknown		19		29	
**Gene fusion**					
BRD4-NUTM1		23		35	
BRD3-NUTM1		2		3	
NSD3-NUTM1		3		5	
Variants		4		6	
Unknown		33		51	
**Symptoms**					
**Yes**		44		68	
**No**		6		9	
**Unknown**		15		23	
Cough		27		42	
Chest pain		18		28	
Shortness of breath		14		22	
Back pain		12		18	
Fever		8		12	
Blood sputum		6		9	
Weight loss		5		8	
**Smoking**					
Yes		16		25	
No		15		23	
Unknown		34		52	

Three statistical risk groups, classified by dissection site and NUT fusion gene type, have been identified. Nonthoracic primary with non-BRD4–NUT fusion confers the best prognosis, followed by nonthoracic primary with BRD4–NUT. Thoracic NMC, regardless of the NUT fusion, has the worst prognosis (9). The prognoses of NMCs of the head and neck have been reported to improve following aggressive initial surgical resection +/− post-operative chemoradiotherapy (CRT) or radiotherapy (RT) (10). However, because NMC of the thorax was diagnosed later, treatments to improve its prognosis have yet to be identified. Thus, we investigated the clinical features of primary thoracic NMC.

### NMC of the thorax

We investigated the clinical features of primary thoracic NMC within the past 15 years (NMC of the thorax investigation) by searching for the terms ‘NUT midline carcinoma lung’ or ‘NUT midline carcinoma mediastinum’ in the PubMed database (https://pubmed.ncbi.nlm.nih.gov/) between 2006 and 2021. The International NMC registry only included data from 1993 to 2007, and because our investigation of NMC of the thorax included cases reported between 2006 and 2021, some cases may have overlapped. Our literature search identified 28 articles describing the clinical features of NMC (11–38). After reviewing these articles describing the clinical features of NMC, we decided to describe 11 clinical features (chief complaint, sex, age at diagnosis, smoking history, primary site, presence or absence of distant metastasis at the time of initial diagnosis, treatment (chemotherapy, RT, surgery), survival after diagnosis and type of genetic mutation). Because few papers mentioned all 11 items, we chose papers describing cases in which at least four of the 11 items were mentioned.

There were 65 cases that met the above requirements: 19 cases from 19 case reports and 46 cases from four review articles (Table 2). Two respiratory physicians (Saiki A. and Sakamoto K.) carefully reviewed the clinical characteristics, age, and primary site of each case to determine whether there were any duplicated cases. If so, one of the cases was subsequently removed from the study cohort. This review outlines the clinical features of NMC of the thorax and the prospects for its treatment.

## Clinical features

The main complaints have not mentioned in the NMC registry. In our NMC of the thorax investigation, the main complaints were cough, chest pain and dyspnea.

Our NMC of the thorax investigation included 34 males, 30 females and one case with sex unknown. As with the NMC registry, no gender bias was observed.

The age at diagnosis ranged from 5 to 70 years, with a median of 30.5 years (mean ± standard deviation, 30.0 ± 15.98) in our NMC investigation. Compared with the NMC registry data, the median age diagnosis increased from 23 to 30.5 years. This age discrepancy was because antibodies were previously only used for younger patients, but in the past 15 years, these were also used for older patients (10).

Smoking history has not been mentioned in the NMC registry. In our NMC of the thorax investigation, smokers and nonsmokers comprised 16 (25%) and 15 (23%) of the patients, respectively, while the smoking status of 34 (52%) patients was unknown.

The primary site was the lungs in 62% of all cases (40/65) in our NMC of the thorax investigation, which was similar to the NMC registry (51%). However, many cases had distant metastases at diagnosis, which could have made it difficult to determine the primary site.

In addition, 44% (29/65) of cases in our NMC of the thorax investigation had distant metastasis at the time of initial diagnosis, which was similar to that in the NMC registry (50%).

The percentage of *BRD4-NUT* fusion genes was 71% in our investigation, which was almost the same as that in the registry (78%). The proportion of fusion genes appears to have remained unchanged during the past 15 years.

Section 6 will discuss two more items: treatment (chemotherapy, RT, and surgery) and survival after diagnosis.

## Biology

BRD4 is a member of the BET (bromodomain and extra-terminal) protein family. It recognizes acetylated lysine in histone and functions as a transcription activator. NUT is a poorly understood protein that recruits and activates the histone acetyltransferase p300. BRD4-NUT is the only oncoprotein that drives NMC growth. Whole-genome and targeted next-generation sequencing (NGS) have revealed that besides the NUT fusion gene, NMCs are genomically stable (39). Massive contiguous regions of chromatin coenriched with BRD4-NUT, p300, and acetylated histones are termed megadomains (MDs). BRD4-NUT binds a small region of acetylated histones and recruits p300 to locally acetylate nearby histones, resulting in further recruitment of BRD4-NUT and p300. This is termed the feed-forward model. The targets of MDs include three stem cell-related transcription factors frequently implicated in cancer: MYC, SOX2 and P63. These three transcription factors are involved in maintaining the stem cell state and preventing cell differentiation (Fig. 3). Notably, the *MYC* gene is directly upregulated by BRD4-NUT. Thus, NMC is considered an *MYC*-driven cancer (39). Eagen and French (39) proposed that MD-mediated upregulation of p63 (ΔNp63) counteracts p53 by inhibiting its transcriptional targets and preventing apoptosis.

## Diagnosis and pathology

NMC is diagnosed by a NUT immunohistochemical (IHC) assay that stains the nuclei of NMC cells and has a specificity of 100% and sensitivity of 87% for the diagnosis of NMC, using monoclonal rabbit antibody to NUT (clone C52B1, Cell Signalling Technology, Danvers, MA, USA). Tumours where >50% of nuclei are IHC-positive on formalin-fixed, paraffin-embedded sections are considered NMC (Fig. 2). Detecting the BRD4-NUT fusion gene by reverse-transcription polymerase chain reaction, fluorescence *in situ* hybridization or NGS is unnecessary (40).

**Figure 1:**
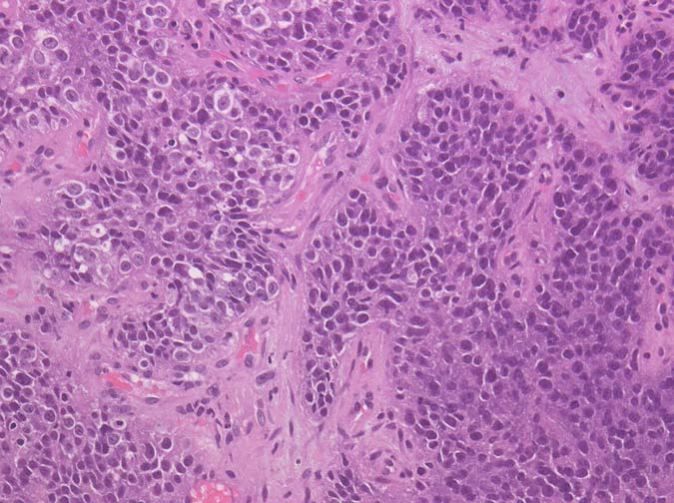
Images are 200x magnification and haematoxylin and eosin stained; proliferation of monomorphic round cells with clear cytoplasm.

Pathologically, NMC is an undifferentiated carcinoma that can exhibit abrupt squamous differentiation in 33–40% of cases. Immunostaining is often positive for epithelial markers, such as cytokeratin AE1/AE3, CK5/6, p63, p40 and EMA (28). The presence of abrupt squamous differentiation can be helpful in distinguishing NC from other poorly differentiated neoplasms or Ewing sarcoma, thymic carcinoma or small-cell carcinoma. However, it is also a characteristic of basaloid squamous cell carcinoma and human papillomavirus (HPV)-associated squamous cell carcinoma of the oropharynx. Viral aetiology, such as HPV or Epstein–Barr virus (EBV), is not associated with NMC. Therefore, it can be used to exclude NMC, which sometimes lacks squamous differentiation. A key characteristic that distinguishes NMC from other poorly differentiated carcinomas is the monomorphism of the cells, which contrasts with the pleomorphism observed in other carcinomas (Fig. 1). Although the histologic features of NMC are inadequate for diagnosis, NUT IHC is recommended to rule out NMC in all undifferentiated carcinomas, with or without squamous differentiation, which have a monomorphic appearance, in the absence of EBV or HPV within the tumour (41). Generally, it is extremely rare to find NMCs with glandular differentiation; thus, these tumours do not need to be tested. The presence of neuroendocrine differentiation or TTF-1 positivity has been reported in NMCs (21,36).

**Figure 2:**
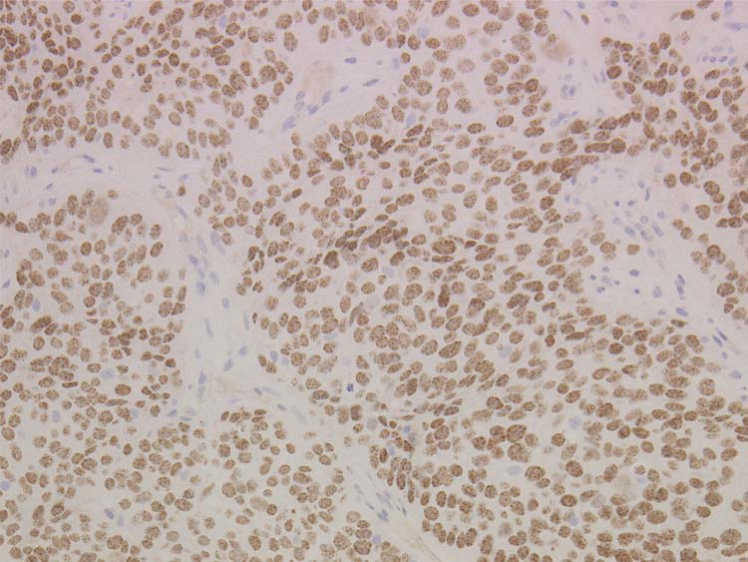
Images are 200x magnification and nuclear protein of the testis (NUT) nuclear immunohistochemical (IHC) stained; >50% of tumour nuclei are positive for NUT nuclear IHC staining.

**Figure 3:**
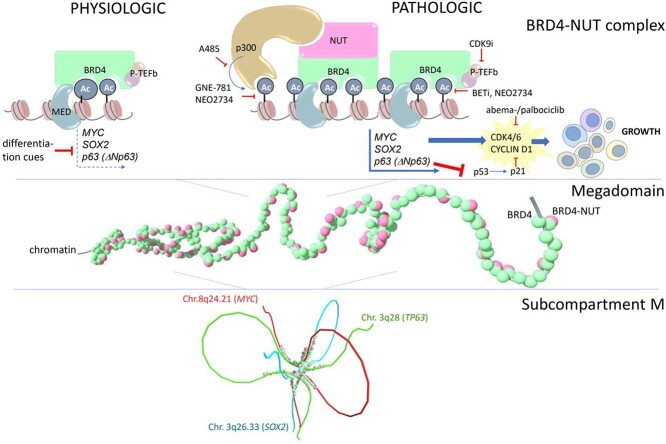
Mechanistic model of how BRD4-NUT drives growth and blocks differentiation in NUT carcinoma. Cited from Eagen KP, French CA. Supercharging BRD4 with NUT in carcinoma. *Oncogene*. 2021;40(8):1396–1408.

## Epidemiology

During the 14 years (1993–2007) of the NMC registry, 141 patients were diagnosed, including 71 primary thoracic NMC patients. Our NMC of the thorax investigation revealed 65 cases of primary thoracic NMC over the past 15 years (2006–2021). Although the data collection method differed, the number of cases of primary thoracic NMC has not increased significantly over the past 15 years. However, the number of cases of head and neck NMC significantly increased after 2012, when the NUT IHC assay became widely available (10).

The incidence of NMC is unknown. According to a report from the Oncology Department of the Princess Margaret Hospital for Children, located in the geographically isolated state of Western Australia, with a catchment population of around 2 million, five NMC patients were identified among all high-grade undifferentiated sarcomas or carcinomas in 0–16-year-olds from 1989 to 2014. The risk of incidence of NMC was estimated at ~0.41 per one million children (0–16 years of age). This incidence rate was independent of the primary site and limited to children (42).

The frequency of NMC among specific cancer types varied and was reported as 0.6% of nonglandular lung carcinomas (1/166) (28), 2.7% of thymic carcinomas (1/37) (43), 3.5% of poorly differentiated mediastinal carcinomas (4/144) (44) and 18% of undifferentiated carcinomas of the upper digestive tract (5/28) (45).

The probability of diagnosis varies greatly between countries. Because the diagnosis requires immunostaining and genetic analysis, many NMC patients worldwide may be undiagnosed.

## Treatment and prognosis

In our NMC of the thorax investigation, the mean survival time after diagnosis was 6.75 months (± standard deviation 4.60). This is almost the same as the International NMC registry (6.5 months). Our method collected data on cases that could be diagnosed and treated before death, while data on cases where patients died without a diagnosis before death were not collected. Therefore, it is likely that the survival time was overestimated.

In our NMC of the thorax investigation, 40% (26/65) of patients were treated by RT. Although the details of the RT dose were unknown, RT was mostly used as a palliative treatment for stage IV patients (21/26). Curative RT, such as CRT or post-operative RT, was used in fewer than five cases.

Only 21.5% (14/65) of patients underwent partial resection or lobectomy. The mean survival period of the 13 patients whose prognosis was known was 8.8 months. While one patient died 3 days after the operation, patients operated on in the early stages of the disease displayed a better prognosis than mean survival time, 6.75 months.

**Table 3:** Clinical trials of BET inhibitor for NMC patients

Clinical trial	Design	Drug	Patients,No	Overall response rate		Median time of study		Toxicity	Recommended dose		Publication		
NCT01587703	3 + 3,escalation	GSK525762	65 pts	NMC cohort *n* = 19		NMC median PFS 2.5 m		G3–4	Safety and PK data		Piha-Paul S. et al, 2018 (41)		
		(molibresib)	CRC *n* = 22	PR *n* = 2				Thrombocytopenia *n* = 24					
			NMC *n* = 19	SD *n* = 8				Nausea *n* = 2					
			CRPC *n* = 9	Non-NMC *n* = 41				Anorexia *n* = 3					
			SCLC *n* = 6	uPR Breast *n* = 1				Vomiting *n* = 1					
			Breast *n* = 5	SD > 4 m CRPC, CRC				Anaemia *n* = 5					
			NSCLC *n* = 2					Br *n* = 3					
			Neuroblast *n* = 1					Fatigue *n* = 3					
			Myeloblast *n* = 1										
NCT02259114	3 + 3,escalation	MK-862	46 pts	42 evaluable pts		2.3 m (0.2–15.4 m)		G3–4	PK data, 80 mg		Lewin 2018 (42)		
		(birabresib)	NUT *n* = 10 (22%)	CR 0				nausea *n* = 1					
			CRPC *n* = 26 (57%)	PR 3 NMC pts (7%)				vomiting *n* = 1					
			NSCLC *n* = 10 (22%)	SD *n* = 25 (60%)				fatigue *n* = 2					
				NMC *n* = 3				anaemia *n* = 11					
				CRPC *n* = 15				thrombocytopenia *n* = 20					
				NSCLC *n* = 7				ALT *n* = 2					
								Acute kidney injury *n* = 1					
								Thrombocytopenia nadir					
								32 d (range = 12–211)					
NCT01987362	3 + 3,escalation	RO6870810	74 pts	PR5	NMC *n =* 2	NMC median PFS94 days		G3–4	dose of 0.65 mg/kg administered		Shapiro GI,2020 (28)		
	(0.03–0.65 mg/kg)		Solid tumour *n* = 47		Solid tumour *n =* 1			fatigue *n* = 7	for 14 of every 21 days.				
			NMC *n* = 8		DLBCL *n* = 2	(range, 15–783 days)		Nausea *n* = 1					
			DLBCL *n* = 19					vomiting *n* = 1					
								Anaemia *n* = 6					
NCT02419417	3 schedules:	BMS 986158	75 pts	1 pt NUT (schedule A)		1 pt with SD,		G3–4	Safety and PK data		Hilton J. et al, 2018 (43)		
	A (5 d on/2 d off)		NUT *n* = 4	279 d, SD		279 d (9.3 months)		Thrombocytopenia *n* = 10 (15%)					
	B (14 d on/7 d off)		Other solid tumour *n* = 71					Fatigue *n* = 1 (1%)					
	C (7 d on/14 d off)							Nausea *n* = 1 (1%)					

CRC = colorectal cancer

CRPC = castration-resistant prostate cancer

NSCLC=Non-Small Cell Lung Cancer

uPR = unconfirmed partial response

There were 14 patients treated with platinum-based chemotherapy. While three patients were treated with carboplatin + paclitaxel, two were treated with cisplatin + paclitaxel, another two were treated with cisplatin + etoposide + bevacizumab and one patient each was treated with carboplatin + docetaxel, cisplatin + docetaxel, carboplatin + nab-paclitaxel. Four patients were misdiagnosed as sarcoma, treated with doxorubicin or ifosfamide. Three of four patients underwent more than four courses and survived >9 months. Chihiro et al. (46) reported that doxorubicin or ifosfamide treatment of NMC had some effect. However, in the past 15 years, the prognosis has not been prolonged, and no effective treatment for NMC has been established.

## Future perspectives

### BET inhibition

BET inhibitors antagonize BRD4 and inhibit BRD4-NUT fusion. Currently, over 15 BET inhibitors are in the early phases of clinical trials (47) (Table 3). In a phase I/II study of molibresib (GSK525762) administered to 19 patients with NMC, two achieved PR and eight showed a stable disease (SD). Four patients who were treated for >6 months had nonthoracic NMC. The two patients who achieved PR were BRD3-NUT fusion gene cases (48). In a phase I study of birabresib administered to 10 patients with NMC, three (30%) achieved PR and three (30%) showed SD. The three PR patients were nonthoracic primary BRD3-NUTM1 fusion gene cases (49). In a phase I study of RO6870810, of the eight patients with NMC who were treated with RO6870810, two (25%) achieved PR, five (63%) showed SD and one (13%) showed a progressive disease (PD). The two patients who achieved PR had primary thoracic NMC with BRD3-NUT and NSD-NUT (50). In a study of BMS-986158, four NUT patients were treated with BMS-986158. One showed tumour shrinkage and 9-month clinical benefit on BMS-986158 and was a BRD3-NUT fusion case (51).

The results of the clinical trials discussed above suggest that NMCs with non-BRD4-NUT fusions may have different biological characteristics that make them more likely to be sensitive to specific BET inhibitors. Unfortunately, patients showing PR were unable to continue in these trials because of dose-limiting toxicity or the development of resistance.

BET proteins are crucial for the functioning of every cell in the body, and BET inhibitors are generally unable to selectively target cell types, targeting not only BRD4 but also BRD2 or BRD3. If BET inhibitors were able to selectively target cells, this would substantially reduce the side effects. On the other hand, the function of NUT protein is unknown and is only expressed in the testis. Thus, NUT-targeted drugs may show reduced side effects. Fertility defects in male patients are expected, but this can be addressed using assisted reproductive technology. Furthermore, the blood–testis barrier can prevent the spread of NUT-target drugs to the testes. Although the protein structure of NUT requires further elucidation, the use of a drug that targets NUT is promising for NMC treatment (39,52).

Proteolysis targeting chimeras (PROTACs) are also a promising treatment for NMC. PROTACs are hybrid drugs that bind to the disease-causing protein and are linked to a ubiquitin-adding enzyme that ubiquitinates and degrades the disease-causing protein. Conventional drugs are only effective while they are bound to the target protein; therefore, they need to maintain their binding. In contrast, PROTACS irreversibly degrade the disease-causing protein after a single binding event. However, binding may be limited by their large size and, thus, limited bioavailability. A trial of ARV-110 (a PROTAC targeting androgen receptors) in patients with metastatic castration-resistant prostate cancer is currently in progress (39,52).

### P300 inhibition

NEO2734 targets the BRD4-NUT-p300 axis to synergistically inhibit both BRD4-NUT and p300. It was significantly superior in prolonging the survival of mouse xenograft models compared with BET inhibitors (39,53).

### Histone deacetylase inhibition

The inhibition of histone deacetylase (HDAC) activity is predicted to spread acetylation across the genome, including regions not occupied by MDs. Thus, HDAC inhibitors should result in BRD4-NUT, which binds to acetylated histones, spreading to areas of newly acetylated chromatin. Because there are limiting quantities of p300, relative depletion of p300 in MDs and MD function deteriorates, resulting in the loss of expression of pro-growth MD target genes. The clinical benefits of HDAC (vorinostat) have been confirmed (54,55).

CUDC-907 is a dual HDAC and phosphoinositide 3-kinase inhibitor. A phase I study of CUDC-907 (NCT02307240) was conducted for NMC patients. However, HDAC inhibitors display poor efficacy in solid tumours compared with haematological malignancies. This is thought to be due to the poor pharmacokinetics of HDAC inhibitors, which are unable to reach therapeutic concentrations *in vitro* (56).

### Cyclin-dependent kinase 9 inhibition

Because cyclin-dependent kinase (CDK) 9 inhibition selectively kills NMC cells, Bragelmann et al. (57) suggested CDK9 inhibition as a treatment for NMC.

### CDK4/6 inhibition

*MYC* is well known to promote cancer growth by upregulating and activating CDK4/6 and cyclin D1. Liao et al. (58) discovered that CDK4/6 inhibitors synergize with BET inhibitors in NMC. CDK4/6 inhibitors are in use and effective in treating oestrogen-positive breast cancer.

## Conclusion

NMC is an extremely rare cancer, and it is thought that many patients remain undiagnosed. As more patients become diagnosed and are given promising treatments, it is hoped that a successful treatment will be established.

## Conflict of interest statement

None declared.
